# Hyponatremia after COVID-19 is frequent in the first year and increases re-admissions

**DOI:** 10.1038/s41598-023-50970-z

**Published:** 2024-01-05

**Authors:** Betina Biagetti, Adrián Sánchez-Montalvá, Albert Puig-Perez, Isabel Campos-Varela, María Florencia Pilia, Emilie Anderssen-Nordahl, Didac González-Sans, Marta Miarons, Rafael Simó

**Affiliations:** 1 Endocrinology Department, Diabetes and Metabolism Research Unit, Vall d’Hebron University Hospital and Vall d’Hebron Research Institute (VHIR), Universidad Autónoma de Barcelona, Barcelona, Spain; 2 Infectious Diseases Department, Vall d’Hebrón University Hospital, Global Health Program from the Catalan Health Institute (PROSICS), Universitat Autònoma de Barcelona, Barcelona, Spain; 3 Mycobacterial Infection Study Group from the Spanish Society of Clinical Microbiology and Infectious Diseases (GEIM-SEIMC), Barcelona, Spain; 4 Infectious Diseases Netword Biomedical Research Center (CIBERINFEC), Instituto de Salud Carlos III, Madrid, Spain; 5 Liver Unit, Vall d’Hebron Hospital Universitari, Vall d’Hebron Institut of Research (VHIR), Vall d’Hebron Barcelona Hospital Campus, Universitat Autònoma de Barcelona, Barcelona, Spain; 6 Centro de Investigación Biomédica en Red de Enfermedades Hepáticas y Digestivas (CIBERehd), Instituto de Salud Carlos III, Madrid, Spain; 7 Pneumology Department, Vall d’Hebron University Hospital, Department of Medicine, Autonomous University of Barcelona (UAB), Barcelona, Spain; 8 Vall d’Hebron Research Institute (VHIR), Barcelona, Spain; 9 Department of Clinical Pharmacology, Vall d’Hebron University Hospital, Vall d’Hebron Barcelona Hospital Campus, Barcelona, Spain; 10 Systemic Autoimmune Diseases Unit, Internal Medicine Department, Hospital Universitario Vall d’Hebron, Vall d’Hebron Barcelona Hospital Campus, Barcelona, Spain; 11 Pharmacy Department, Vall d’Hebron Hospital Universitari, Vall d’Hebron Barcelona Hospital Campus, Barcelona, Spain

**Keywords:** Viral infection, Prognostic markers

## Abstract

Hyponatremia on admission has been related to worse outcomes in patients with COVID-19 infection. However, little is known about the frequency and the associated risk factors of hyponatremia after COVID-19 discharge. We performed an observational 24-month follow-up study of patients admitted during the first COVID-19 wave. Kaplan–Meier curves and Cox proportional hazard models were used to assess the main variables in predicting hyponatremia on follow-up (HYPO-FU). A total of 161 out of 683 (24.4%) developed HYPO-FU. The group with HYPO-FU comprised of more men [(62.3%) vs. (49.2%); *p* < 0.01], older [65.6 ± 18.2 vs. 60.3 ± 17.0; *p* < 0.01] and more frequently re-admitted [(16.2%) vs. (3.8%); *p* < 0.01). The rate of HYPO-FU was higher in the first year 23.6 per 100 individuals per year. After Cox regression analysis, the independent risk factors of HYPO-FU were diabetes [OR 2.12, IC 95% (1.48–3.04)], hypertension [OR 2.18, IC 95% (1.53–3.12)], heart failure [OR 3.34, IC 95% (1.72–6.48)] and invasive ventilation support requirement [OR: 2.38, IC 95% (1.63–3.50)]. To conclude, HYPO-FU was frequent in the first year after COVID-19 infection, and the risk was higher in older men with comorbidities, increasing rehospitalisation. Further studies aimed at evaluating the beneficial effects of correcting hyponatremia in these patients are warranted.

## Introduction

Up to 30% of hospitalized patients develop hyponatremia^1–4^ and it has been related to worse outcomes such as extended length of hospital admission^5^, longer intensive care unit (ICU) stay and mortality^6^.

Pneumonia, has been associated with increased risk of hyponatremia, which is a worse prognostic marker^7^, and has been included in some scores of pneumonia’s severity^8,9^.

The mechanisms causing hyponatremia in patients with pneumonia are far from being elucidated. Systemic inflammation^10^, alveolar perfusion-ventilation mismatch^11^ and stress-related antidiuretic hormone (ADH) release^12,13^ have been involved as pathogenic factors. In addition, the overproduction of B-natriuretic factor^14^, a decrease in renal water clearance, and the concomitant use of natriuretic drugs might also contribute to hyponatremia.

Particularly, hyponatremia at admission for coronavirus disease 2019 (COVID-19) it is around 20–30% and has been related to higher 30-day mortality and adverse outcomes^15–17^.

Long COVID or post-COVID-19 syndrome is the term used to describe the long-term effects/sequelae of COVID-19^18,19^. Some studies report persistent symptoms after non-severe COVID-19^20^ and the presence of signs and symptoms affecting quality of life in up to 90%^21^, irrespective of the viral strain^22^. Increasing numbers of studies on post-acute and prolonged symptoms of COVID-19 have been published worldwide, even showing changes in brain structure, mainly in the limbic system ^23^. Moreover, outpatient care for these patients requires a multidisciplinary healthcare approach in specialized post-COVID units^24–26^. However, no study so far has investigated the incidence and prevalence of hyponatremia. After discharge from hospital due to admission for COVID-19, that is, in outpatient follow-up.

Our hypothesis was that patients discharged after COVID-19 infection are likely to develop hyponatremia also in the follow-up. In order to verify this hypothesis, we have determined the incidence and prevalence of hyponatremia after discharge in those patients who were admitted due to SARS-CoV-2 infection. In addition, the associated risk factors and readmission rate have been investigated.

## Material and methods

### Study design and participants

This study was approved by the Ethics Committee of the Vall d’Hebron University Hospital (number: PR(AG)229-2021), and conducted according to the declaration of Helsinki and Good Clinical Practice Guidelines. In accordance with the national legislation and the institutional requirements the patient consent was waived because the study was retrospective, containing de-identified data.

Following the Strobe rules^27^, we performed an observational, retrospective study with prospective follow-up of patients admitted with COVID-19 infection from March 2020 to April 2020 at the adult emergency department of Vall d’Hebron University Hospital during the first pandemic wave. We benefit of a redcap database that was specifically designed to study COVID-19 outcomes in the first pandemic wave. The exposure factor was to have been admitted with COVID-19 infection. The inclusion criteria were, adult patients without age restriction, with a positive nasopharyngeal PCR swab test for SARS-CoV, respiratory symptoms (e.g. cough, dyspnoea) and a blood test with sodium levels on admission. In case of multiple admissions during the study inclusion period, we considered the first emergency room (ER) data as the baseline.

### Clinical evaluation, tests and management

Vital parameters including body temperature, respiratory rate, oxygen saturation, heart rate, blood pressure, blood test analysis, chest radiography and clinical management were performed in every patient following the standard of care of our hospital.

### Study variables and outcome

Data about baseline characteristics (e.g. age, sex, body mass index (BMI), vital signs, comorbidities (e.g. hypertension, type 2 diabetes (T2DM), ischemic cardiopathy, heart failure (HF), previous pneumological diseases) and blood test including natremia were collected at admission.

Further variables including serial blood test, ventilation support, ICU admission, length of hospital stay, outcomes at discharge such as transfer to other hospital or mortality up to discharge were recorded by a multidisciplinary staff in a redcap database specifically designed to study COVID-19 outcomes. The REDcap database had drop-down menus and entry data restrictions according to each parameter as a quality control measure. Subsequently, a computer search was performed for rehospitalization, blood tests after discharge and death. Catalunya has a centralized shared medical system (CSMS) thus, for all public services including the primary care services the discharges, admissions, tests including blood test with sodium levels and death are centrally available. In addition, these outcomes can be automatically drop to a database without manual entry (see supplementary material).The follow-up of outcomes after discharge (i.e. hyponatremia after discharge, re-admission, death) was traced up to the 30th of September 2022.

Hyponatremia on follow-up (HYPO-FU) was established when at least one blood sodium level below 135 mmol/L was detected in the informatic tracing system after discharge.

### Statistical analysis

Categorical variables are expressed as number (n) and percentage (%) and compared using the Chi-Square test or with Fisher’s exact test when at least one of the expected frequencies was less than 5. Continuous variables were expressed in mean and standard deviation (SD), and comparison between two groups was performed using Student’s *t*-test or the nonparametric Mann–Whitney U-test according to variable distribution.

The exposure factor was had been admitted with COVID-19 infection in the first wave, the primary outcome was HYPO-FU and the secondary outcome were readmissions and death in the follow-up.

The “a priori” sample size to have a confidence level of 95% regarding the prevalence of hyponatremia in follow-up was 289 individuals. For this we presumed that at maximum the prevalence of hyponatremia in the follow-up would be similar that the prevalence of hyponatremia upon admission during the first wave of for COVID-19, which was around 25%^15–17^.

To avoid confounding factors, both univariate and multivariable models were applied for the comparisons of different groups (i.e. hyponatremia at follow-up versus normonatremia at follow-up).

The HYPO-FU was considered as the main event; patients who died were censored in the date of death. The time to the event was defined as the interval between the hospital discharge and the HYPO-FU event or to the informatic trace (30th of September 2022) of the censoring patients without HYPO-FU (in days). Kaplan–Meier curves were constructed to trace survival (SV). We also used the actuarial method and grouped periods by six months with a descriptive objective. Cox proportional hazard models were used to assess those factors associated with HYPO-FU. First, we compared the raw SV curves using log-rank, Tarone-Ware and Wilcoxon tests. The following variables were tested: (1) Patient’s characteristics: age, gender, BMI; (2) Comorbidities: diabetes, hypertension, ischemic cardiopathy, stroke, pneumopathy, hepatopathy or cancer; (3) Severity: hyponatremia at admission, ICU admission, invasive ventilation, corticosteroids and length of stay. These analyses were performed unadjusted and then carried out with a confounder’s adjustments. A stepwise Cox regression analysis was then executed. The final selected model was evaluated by its predictive power using Harrell’s C concordance statistic. Results are reported as mean incidence rate hazard ratios (HR) and 95% confidence intervals (CI). A two-sided *P*-value of < 0.05 was considered significant. All statistical tests were performed using the STATA 16 package.

### Ethical approval

The study was conducted according to the mandates in the Declaration of Helsinki, and the Ethics Committees of the Vall d’Hebron University Hospital. Date 2020 and amended 2021 (number: PR(AG)229–2021).

## Results

### Baseline characteristics

Of a total of 1.287 records of patients admitted in the ER in the first wave of the pandemic, 865 met the inclusion criteria: 471 (54.5%) were male, mean age of 64.6 ± 17.4 years, with 289 (33.4%) older than 75 years, and BMI of 29.1 ± 5.3 kg/m^2^ without statistical differences between gender Table 1. Men had more baseline comorbidities, diabetes [108 (23.0%) vs. 67 (17.0%) *p* < 0.01], ischemic cardiopathy [59 (12.5%) vs. 22 (5.6%); *p* < 0.01], nephropathy [67 (14.3%) vs. 35 (8.9%) *p* < 0.01], more frequently presented hyponatremia on admission [117 (37.6%) vs. 119 (30.2%) *p* = 0.02], required ICU admission [117 (25.5%) vs. 76 (17.3%); *p* = 0.03] and a higher percentage died during admission [113 (24.0%) vs. 69 (17.5%), *p* = 0.02] compared with women. The patients who died, had more hyponatremia on admission without reaching statistical significance [110 (19.3%) vs. 72 (24.3%); *p* = (0.09)].

**Table 1:** Baseline characteristics and outcomes by gender.

	All	Men	Women	*p* value
N (%)	865 (100)	471 (54.5)	394 (45.5)	–
Age: years, mean (SD)	64.6 (17.4)	64.7 (16.7)	64.4 (18.4)	0.77
Older than 75 years old, n (%)	289 (33.4)	161 (34.2)	128 (32.5)	0.70
BMI: kg/m^2^ mean (SD)^a^	29.1 (5.3)	28.8 (5.0)	29.4 (5.7)	0.26
Obesity, n (%)^a^	143 (38.8)	67 (35.1)	76 (42.7)	0.19
Diabetes, n (%)	175 (20.3)	108 (23.0)	67 (17.0)	0.03
Hypertension, n (%)	432 (50.0)	239 (50.9)	193 (49.0	0.59
Ischemic cardiopathy, n (%)	81 (9.4)	59 (12.5)	22 (5.6)	0.01
Stroke, n (%)	91 (10.5)	46 (9.8)	45 (11.4)	0.44
Pneumopathy, n (%)	184 (21.4)	110 (23.5)	74 (18.8)	0.10
Hepatopathy, n (%)	31 (3.6)	16 (3.4)	15 (3.8)	0.69
Nephropathy, n (%)	102 (11.9)	67 (14.3)	35 (8.9)	0.01
Cancer, n (%)	108 (12.5)	67 (14.3)	41 (10.8)	0.09
Length of Stay days, mean (SD)	11.8 (15.0)	12.7 (16.3)	10.7 (13.1)	0.06
Hyponatremia at admission	296 (34.2)	177(37.6)	119 (30.2)	0.02
ICU admission, n (%)	193 (22.7)	117 (25.5)	76 (19.3)	0.03
Death at admission, n (%)	182 (21.0)	113 (24.0)	69 (17.5)	0.02
Re-admission, n(%)^b^	45 (6.6)	24 (6.7)	21 (6.5)	0.90
Follow-up: days, mean (SD)	743.0 (371.0)	727.1 (387.4)	760.2 (348.3)	0.06

^a^Reported in 369 patients, BMI: body mass index, SD: standard deviation.

^b^Reported in 678 patients (182 patients had died and 5 patients had the residency out of Catalunya or without SS).

### Hyponatremia on follow-up

A total of 182 patients died on admission; thus, follow-up was carried out in 683 patients. Overall, 167 (24.5%) had hyponatremia after discharge on the follow-up (Table 2**).** Patients with HYPO-FU were more frequently men [104 (62.3%) vs. 254 (49.2%); *p* < 0.01], older [65.6 ± 18.2 vs. 60.3 ± 17.0; *p* < 0.01], with more comorbidities such as diabetes [57 (34.7%) vs. 66 (12.8%); *p* < 0.01], hypertension [103 (61.1%) vs. 191 (37.1%); *p* < 0.01], ischemic cardiopathy [25 (14.0%) vs. 24 (4.4%); *p* < 0.01], heart failure [11 (6.8%) vs. 5 (1.0%); *p* < 0.01], nephropathy [34 (20.5) vs. 21 (4.1%), *p* < 0.01], cancer [25 (15.0%) vs. 35 (6.8%); *p* < 0.01] and presented more frequently with hyponatremia on admission [77 (46.1%) vs. 147(28.5%); *p* 0.01] compared with patients without HYPO-FU. Patients with HYPO-FU also had had more: length of stay (19.4 ± 19.3 vs. 10.4 ± 12.0 days; *p* < 0.01), ICU admission [40 (24.5%) vs. 60 (11.8%); *p* < 0.01], invasive ventilation [39 (23.9%) vs. 59 (11.6%); *p* < 0.01], corticosteroids requirement [40 (28.0%) vs. 47 (10.4%); *p* < 0.01] and more rate of re-admissions [26 (16.2%) vs. 19 (3.8%); *p* < 0.01].

**Table 2:** Characteristics of patients with hyponatremia in the follow-up.

	All	Hyponatremia NO	Hyponatremia YES	*p* value
	N = 683	N = 516	N = 167	
Male gender, n (%)	358 (52.4)	254 (49.2)	104 (62.3)	0.01
Age: years, mean (SD)	61.2 (17.4)	60.3 (17.0)	65.6 (18.2)	0.01
Older than 75 years old, n (%)	171 (25.0)	113 (21.9)	58 (34.7)	0.01
BMI: kg/m^2^ mean (SD)	29.0 (5.7)	28.8 (5.2)	29.9 (6.6)	0.15
Obesity, n (%)	114 (38.8 )	82 (37.4)	32 (42.7)	0.42
Diabetes, n (%)	123 (18.0)	66 (12.8)	57 (34.7)	0.01
Hypertension, n (%)	294 (43.1)	191 (37.1)	103 (61.1)	0.00
Ischemic cardiopathy, n (%)	48 (7,0)	24 (4.4)	24 (14.0)	0.01
Heart failure, n (%)	16 (2.4)	5 (1.0)	11 (6.8)	0.01
Stroke, n (%)	58 (8.5)	40 (7.8)	18 (10.8)	0.21
Pneumopathy, n (%)	118 (17.4)	83 (16.1)	35 (21.3)	0.14
Hepatopathy, n (%)	23 (3.4)	14 (2.7)	9 (5.4)	0.09
Nephropathy, n (%)	55 (8,1)	21 (4.1)	34 (20.5)	0.01
Cancer, n (%)	60 (8.8)	35 (6.8)	25 (15.0)	0.01
Length of Stay days, mean (SD)	12.4 (15.0)	10.4 (12.0)	19.4 (19.3)	0.01
Hyponatremia at admission	224 (32.8)	147 (28.5)	77 (46.1)	0.01
ICU admission, n (%)	100 (14.9)	60 (11.8)	40 (24.5)	0.01
Invasive ventilation, n (%)	98 (14.6)	59 (11.6)	39 (23.9)	0.01
Corticoids, n (%)	87 (14.7)	47 (10.4)	40 (28.0)	0.01
Death follow-up, n (%)	45 (6.7)	31 (6.1)	14 (8.4)	0.30
Re-admission, n(%)	45 (6.8)	19 (3.8)	26 (16.2)	0.01

BMI: body mass index, SD: standard deviation.

### Survival analysis

**Figure 1:**
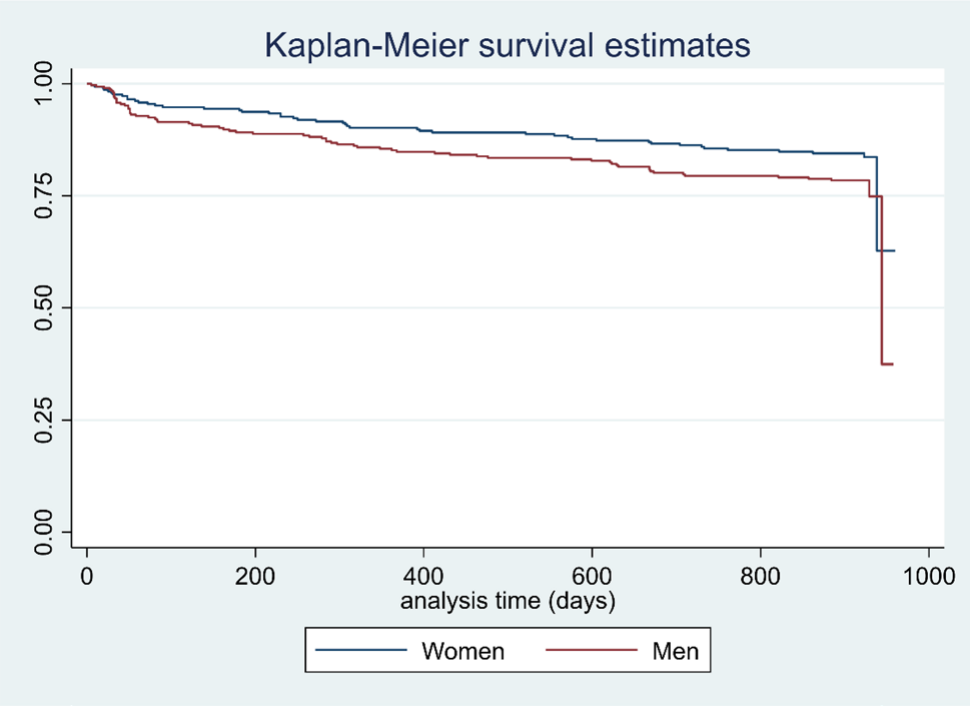
Kaplan–Meier Survival curves by gender. The figure shows the Kaplan Meier Survival curves by gender which was lower in men, logrank test *p* = 0.03.

A total of 167 patients had HYPO-FU (24.5%), 104 men (62.3%) and 63 women (37.7%) *p* = 0.02. The Fig. 1 shows the Kaplan Meir survival curve of HYPO-FU by gender and that it was lower in men, logrank test *p* = 0.03. Table 3 shows the SV failure and HR by the actuarial method grouping by 6 months. The cumulative SV (time without HYPO-FU) at 12 months is 81.7%, meaning that the probability of HYPO-FU before 12 months is 18.3% (cumulative failure). The highest mean incidence rate hazard of HYPO-FU appears in the first 2 semesters: 0.0140 and 0.0197 respectively. In other words, 16.8% and 23.6% patients (expressed in number of cases by 100 patients and year), presented hyponatremia in the first year of follow-up.

**Table 3:** Survival, failure and incidence rate HR by actuarial method grouping by 6 months.

Interval months	Risk	Hypo_Na	Survival	[95% CI]	Cum failure	Hazard	[95% CI]
0–6	683	60	0.9195	0.8959–0.9379	0.0805	0.0140	0.0102–0.0178
6–12	592	69	0.8169	0.7849–0.8446	0.1831	0.0197	0.0150–0.0244
12–18	525	8	0.8044	0.7717–0.8329	0.1956	0.0026	0.0008–0.0043
18–24	515	19	0.7747	0.7405–0.8050	0.2253	0.0063	0.0034–0.0091
24–30	496	7	0.7596	0.7241–0.7911	0.2404	0.0033	0.0009–0.0057
30–36	213	4	0.7316	0.6869–0.7710	0.2684	0.0063	0.0001–0.0124

The parsimonious and best predictive model (see methods) for HYPO-FU identified 4 variables: presence of hypertension, T2DM, HF, and invasive ventilation but not gender or age. (Table 4).

**Table 4:** Risk factors of Hyponatremia in the follow-up by Cox regression analyses.

Variable	HR	[95% CI]	*p*
T2DM	2.12	1.48–3.04	0.00
Hypertension	2.18	1.53–3.12	0.00
Heart failure	3.34	1.72–6.48	0.00
Invasive ventilation	2.38	1.63–3.50	0.00

**Figure 2:**
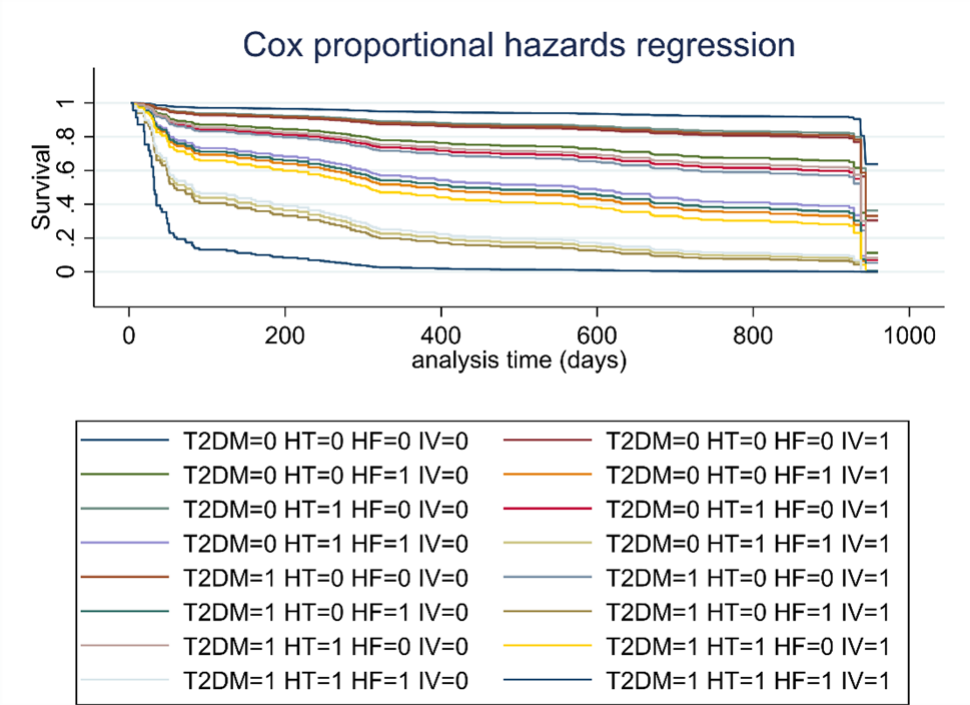
Survival curves according risk factors. The figure shows the survival curves according to the main risk factors: Type 2 diabetes (T2DM), Hypertension (HT), heart failure (HF) and *Invasive ventilation (IV).* The probability of developing a HYPO-FU after the COVID19 episode increased with the number of the selected comorbidities of the model.

Figure 2 shows the Cox survival curve, taking into account the presence of hypertension, T2DM, HF, and invasive ventilation. The probability of developing a HYPO-FU after the COVID19 episode increased with the number of the selected comorbidities of the model.

## Discussion

Our study shows that hyponatremia in the follow-up after COVID infection affects up to 24.5% of patients, over a 2-year period of follow up. Patients with HYPO-FU were more often older men with comorbidities and were more frequently re-admitted. The highest mean incidence rate of HYPO-FU appeared in the first year and the major independents risk factors were, T2DM, HF, hypertension and invasive ventilation requirement. Although hyponatremia on admission was more frequent in the group of patients with HYPO-FU, it was not and independent risk factor, neither gender or age, after adjusting for other variables.

Previous works focused on hyponatremia on admission found worse outcomes not only in patients with pneumonia^28–30^ including COVID-19^10,15,17,31–36^ but also in other diseases^37–41^ such as, chronic kidney disease^42^, heart failure^43^ and liver cirrhosis^40^. Moreover, a meta-analysis by Corona et al.^44^, which included studies comparing mortality rate in subjects with or without hyponatremia, found a higher risk of mortality in patients with hyponatremia in several conditions, such as, myocardial infarction (RR = 2.83 [2.23–3.58]), heart failure (RR = 2.47 [2.09–2.92]), cirrhosis (RR = 3.34 [1.91–5.83]), pulmonary infections (RR = 2.49 [1.44–4.30]). In our study the group of patients who died on admission, had more prevalence of hyponatremia on admission but, without reaching statistical significance.

However, the major question regarding hyponatremia is whether hyponatremia contributes directly to poor outcomes or is only a marker for severity and progression of underlying comorbidities^44,45^. We have some evidence regarding patients admitted with community acquired pneumonia and hyponatremia at discharge, who had a higher recurrence rate of pneumonia within the first 180 days after hospitalization, compared to patients with normal serum sodium levels at discharge^46^. These results could suggest a direct effect of hyponatremia i.e. influencing immune response or alternatively, be only a clinical marker of patients with more comorbidities.

As stated above, hyponatremia is frequent in community acquired pneumonia, being a risk marker for poor outcomes^7^ and it is included in some scores on the severity of pneumonia^8,9^. The exact mechanisms underlying hyponatremia in patients with pneumonia are not completely understood. The most likely are related to ADH release in the context of systemic inflammation which could be a non-osmotic stimulus for ADH production^10^. In addition, pulmonary inflammation could favour an alveolar perfusion-ventilation mismatch, which could lead to lung vasoconstriction, thus resulting in a decrease in left atrial stretch and triggering ADH secretion^11^. Other potential mechanisms could be ADH-independent associated with concomitant true or relative hypovolemia due to inadequate water intake, systemic vasodilation, extra-renal sodium losses for fever or excess of brain natriuretic peptide^14,47,48^. Likewise, diabetes could promote hyponatremia directly by osmosis in a hyperglycaemic state, but also in normoglycemia due to some degree of hyporeninemic hypoaldosteronism^49^.

Male gender has been found as an independent predictor of the severity of COVID-19^50^ and hyponatremia on admission^31^. By contrast, we found that the patients with more comorbidities had a higher risk of developing HYPO-FU independently of gender, age or previous hyponatremia on admission, thus arguing in favour of hyponatremia as a clinical marker of underlying co-morbidities. The differences between our results in comparison with the studies evaluating hyponatremia on admission, could be attributed to the key role of comorbidities rather than gender as underlying key factors of the associated hyponatremia during follow-up.

Taking into account that sodium is the main cation in the extra cellular fluid (ECF) and reflects the amount of water in the ECF^51^, the maintenance of sodium within normal levels is an essential component of ionic and water homeostasis and obviously, it is vital for survival. Thus, patients with comorbidities are less likely to overcome hydric balance.

With reference to hyponatremia treatment, few studies to date have addressed the issue of the potential direct effect of hyponatremia correction on mortality or other adverse outcomes. A recent meta-analysis showed that hyponatremia improvement decreased overall mortality up to 60% compared to patients without hyponatremia improvement^52^. Nevertheless, this could be the result of better evolution of underlying infection or comorbidities. It is worth mentioning that SGLT2 inhibitors, which increase free water excretion, through glucose-induced osmotic diuresis, do not prevent hyponatremia in T2DM patients^53^. Moreover, the majority of studies have failed to show any improvements in long-term outcomes for mortality and rehospitalization with AVP V2 receptor antagonists in HF^54,55^ or cirrhosis^56^. Thus, the beneficial effect of hyponatremia correction in long-term outcomes and mortality is still unknown. An interventional clinical trial (NCT03557957) to address this specific question in terms of determining the effects on mortality and rehospitalization rate of a targeted correction of plasma sodium concentration in addition to current standard care in hospitalized patients with hyponatremia is currently ongoing^57^.

Our study has some limitations. First, due to the retrospective design there was not a scheduled blood test during follow-up in all patients. Second, in close relationship with the retrospective nature of the study, patients with more severe pneumonia, as well as with other comorbidities could be actively followed, thus resulting in more blood testing. The strength of this study lies in the number of patients included, the accurately baseline variable registration and the prospective follow-up. In addition, these results open a new line of research in patients affected by long COVID.

In conclusion, hyponatremia in the follow-up of patients after COVID-19 discharge was frequent in the first year, in older men with comorbidities and it increased the rehospitalisation rate. The independent associated risk factors were diabetes, hypertension, HF and previous invasive ventilation support requirement. Further studies aimed at evaluating the beneficial effects of correcting hyponatremia in these patients are warranted.

### Supplementary Information

Supplementary Information.

## Data Availability

The datasets generated during and/or analysed during the current study are not publicly available but are available from the corresponding author on any reasonable request.
