# Supplementary material for: Hyponatremia after COVID-19 is frequent in the first year and increases re-admissions
Source: Sci Rep. 2024 Jan 5;14:595. doi: 10.1038/s41598-023-50970-z (PMC10770325; doi:10.1038/s41598-023-50970-z)
Supplement: Supplementary file 1 — Supplementary Information. [file 41598_2023_50970_MOESM1_ESM.pdf]

Supplementary material

Hyponatremia after COVID-19 is frequent in the first year and increases re-admissions.

Betina Biagetti*[1] Adrián Sánchez-Montalvá [2,3,4] Albert Puig-Perez[1], Isabel Campos-Varela [5,6] María Florencia Pilia[7,8], Emilie Anderssen-Nordahl[9], Didac González-Sans[10], Marta Miarons [11], Rafael Simó*[1].

Computer search by the statistic staff:

There were 1287 patients registered in REDCap which are identified by an internal hospital code.

This code was used in Ex data (a centralized shared medical system) to identified date and blood test of this patients from patient's discharge to 30th of September 2022. Sodium levels results, readmission and death were extracted linked with the date and with the internal code.

The data were coded quantitatively (sodium value) and/or binary (hyponatremia yes/no readmission yes/no and death yes/no).

Some patients had more than one row, because they had several tests with sodium levels or readmissions in the follow-up.

Subsequently, they merged the two databases (ex_data and REDCap) and we used the merged database to perform the statistical analysis.
